# Role of connexins in human congenital heart disease: the chicken and egg problem

**DOI:** 10.3389/fphar.2013.00070

**Published:** 2013-06-03

**Authors:** Aida Salameh, Katja Blanke, Ingo Daehnert

**Affiliations:** Clinic for Pediatric Cardiology, Heart Centre, University of LeipzigLeipzig, Germany

**Keywords:** connexin, gap junction, cardiac malformations, cardiac morphogenesis, mutation

## Abstract

Inborn cardiac diseases are among the most frequent congenital anomalies and are the main cause of death in infants within the first year of age in industrialized countries when not adequately treated. They can be divided into simple and complex cardiac malformations. The former ones, for instance atrial and ventricular septal defects, valvular or subvalvular stenosis or insufficiency account for up to 80% of cardiac abnormalities. The latter ones, for example transposition of the great vessels, Tetralogy of Fallot or Shone’s anomaly often do not involve only the heart, but also the great vessels and although occurring less frequently, these severe cardiac malformations will become symptomatic within the first months of age and have a high risk of mortality if the patients remain untreated. In the last decade, there is increasing evidence that cardiac gap junction proteins, the connexins (Cx), might have an impact on cardiac anomalies. In the heart, mainly three of them (Cx40, Cx43, and Cx45) are differentially expressed with regard to temporal organogenesis and to their spatial distribution in the heart. These proteins, forming gap junction channels, are most important for a normal electrical conduction and coordinated synchronous heart muscle contraction and also for the normal embryonic development of the heart. Animal and also some human studies revealed that at least in some cardiac malformations alterations in certain gap junction proteins are present but until today no particular gap junction mutation could be assigned to a specific cardiac anomaly. As gap junctions have often been supposed to transmit growth and differentiation signals from cell to cell it is reasonable to assume that they are somehow involved in misdirected growth present in many inborn heart diseases playing a primary or contributory role. This review addresses the potentional role of gap junctions in the development of inborn heart anomalies like the conotruncal heart defects.

## INTRODUCTION

Congenital heart defects belong to the most frequent inborn anomalies with a prevalence of about 10 out of 1000 live births (Schwedler et al., 2011). The pathology ranges from moderate defects like atrial septal defects (ASDs) or ventricular septal defects (VSDs), patent ductus arteriosus or pulmonary valve stenosis up to severe and complex heart diseases like Morbus Fallot, hypoplastic right or left heart syndrome, transposition of the great arteries (TGA), Ebstein’s malformation or Truncus arteriosus communis (TAC). Depending on the severity of the heart disease clinical course varies from spontaneous recovery up to the necessity for multiple surgical interventions and a life-long medical treatment. Nowadays, as pediatric surgery achieved a high level of standard most of the children have a good prognosis with respect to life expectancy and most of them may reach adulthood.

Interestingly, inborn cardiac diseases tend to occur with increased frequency in familial clusters with a significantly elevated risk for cardiac malformations in first degree relatives, siblings or twins. Regarding the latter ones: the prevalence of an inborn heart disease, if either of the twins is affected, is considerably higher for a monozygotic twin sibling compared to a dizygotic twin sibling (Mitchell et al., 1971; Wang et al., 2012). Additionally, if in a monozygotic twinship both twins exhibit any congenital heart defect, then with over 90% incidence both will have the same heart malformation (Seides et al., 1979).

However, up to present the precise cause for the development of an inborn heart disease remains unknown. There are several reports about the influence of exogenous noxa, chromosomal aberration, and genetic and environmental factors, but even for inborn cardiac defects which occur together with well-defined gene variants, for instance the Holt–Oram syndrome or Noonan syndrome (the so-called Mendelian syndromes) or are associated with chromosomal anomalies (for instance Trisomie 18 or 21), the penetrance of the cardiac defect does not reach 100%, and also the type of cardiac defect (i.e., ASD or VSD, pulmonary valve stenosis, etc.) is not identical within a specific syndrome (van der Bom et al., 2011). Thus, it seems obvious that inborn cardiac diseases have a multifactorial genesis and the etiology of most cases remains uncertain (Nora, 1968; Blue et al., 2012).

In the last decade, increasing evidence appeared that cardiac gap junction proteins, the connexins (Cx), might have an impact on cardiac anomalies. In the heart, mainly three of them (Cx40, Cx43 and Cx45) are differentially expressed with regard to temporal organogenesis and spatial distribution. The connexins are named after their molecular weight and the species in which they occur, i.e., the human Cx43 (hCx43) has a molecular weight of approximately 43 kDa (Eiberger et al., 2001).

**FIGURE 1:**
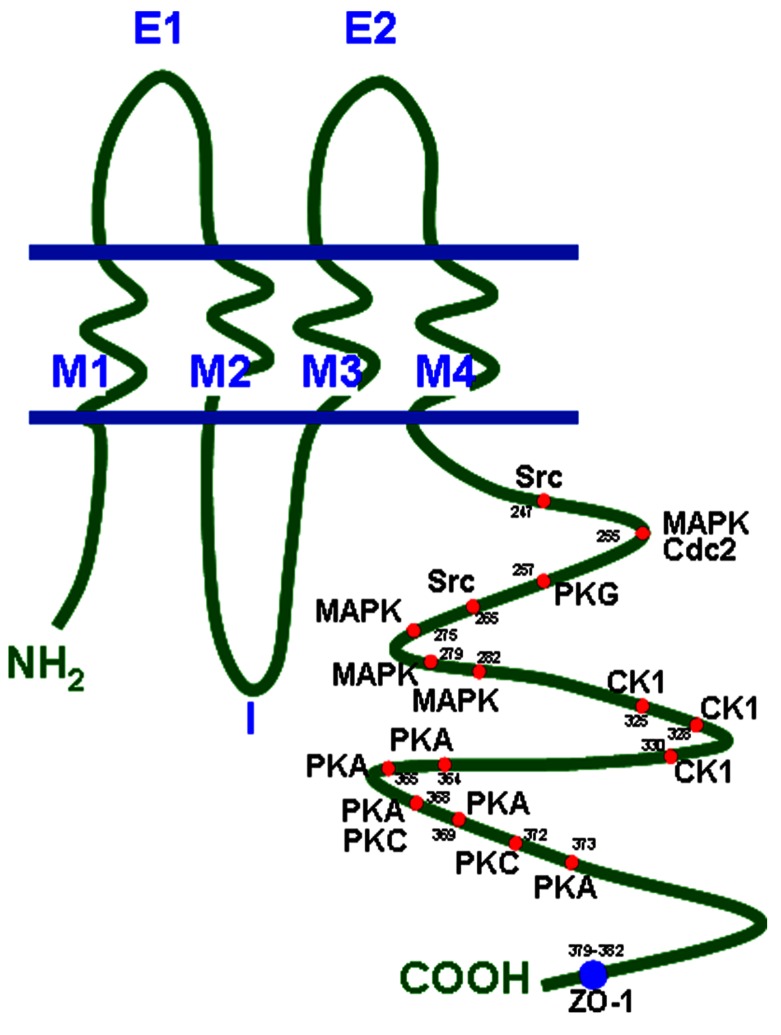
**Connexin topology with phosphorylation sites and ZO-1 binding region.** Cdc2, cyclin dependent kinase 2; CK1, casein kinase 1; MAPK, mitogen-activated protein kinase; PKA, protein kinase A; PKC, protein kinase C; PKG, protein kinase G; ZO-1, zonula occludens protein 1.

Connexin proteins form gap junction channels, which can be considered as pore-like channels permeable for cations, anions, and small molecules with low molecular weights (Simpson et al., 1977). A complete dodecameric gap junction channel is composed of two hemichannels (connexons) contributed by two adjacent cells and one hemichannel consists of six protein subunits. The connexins are transmembrane proteins with four transmembrane domains (M1–M4), two extracellular loops (E1, E2), one intracellular loop, and the N- and C-terminus at the cytoplasmic side of the cell (Unwin and Zampighi, 1980). The C-terminus, which is the most variant part of a connexin differs in length and amino acid sequence and also contains various consensus sequences for a number of protein kinases. For the Cx43 protein it is known that protein kinase A and C (PKA and PKC), mitogen-activated protein kinase, cyclin kinase 1, and some more precisely regulate Cx43 turnover as well as gap junctional communication (Lampe, 1994; Kwak et al., 1995; TenBroek et al., 2001; Polontchouk et al., 2002; Lampe and Lau, 2004). Its protein structure with its phosphorylation sites is presented in **Figure 1** (according to Giepmans, 2004). Besides Cx43 phosphorylation sites have also been identified within the C-terminal tail of Cx40 and Cx45 which alter their channel permeability and electrophysiological properties (Lampe and Lau, 2004).

It is known that some cardiac connexins, particularly Cx43, have considerable short half-lives of about 2–3 h (Darrow et al., 1995; Beardslee et al., 1998). Such a short half-life suggests that the permanent adaptation of the communication structure (i.e., the gap junctions) to environmental conditions might be of pronounced importance for cardiac cells and also might point towards a large functional relevance of connexins in ensuring cardiac development and function. As an example, it was shown that Cx43 changed its localization quickly after starting cyclic mechanical stretch (within 24 h) from a circumferential distribution to an accentuation at the cell poles; similarly polar accentuation was lost after discontinuation of stretch (Salameh et al., 2010). This shows how cells adapt their communication structure (given by the geometric distribution of Cx43) to an external factor (cyclic mechanical stretch) with a time span of 8–12 times the half-life time of Cx43, which is a reasonable period to assume a complete turnover of the protein.

In the human *adult* heart, the spatial distribution of the three connexins (Cx40, Cx43, and Cx45) is not uniform, as they are localized in specialized compartments: atrial myocytes express both Cx40 and Cx43 at similar levels whereas Cx45 is much lower in the atria. In the ventricles, the dominant connexin is Cx43 and in the sinus node and the conduction system of the heart both Cx40 and Cx45 are found (Davis et al., 1995). This non-uniform distribution is the basis for a regular impulse formation, a normal electrical conduction and coordinated synchronous heart muscle contraction. Furthermore, in order to allow regular rhythmic activation of the heart the connexins are typically assembled to gap junction channels at the intercalated disks which are located at the cell poles (Peters et al., 1994; van Rijen et al., 2006). This facilitates conduction of the action potential from cell to cell along the cell axis (Dhein et al., 2011). However, connexins also exist at the lateral border of the cells but at lower abundance. This results in a fast conduction along the fiber axis and a slower conduction transverse to the axis (Dhein et al., 2011).

Besides their functions in the adult heart, the connexins are also most important for the normal embryonic heart development.

Studies in mice revealed that Cx40 is initially expressed in the atrial compartments and later in both ventricles, whereas its expression is turned up earlier in the left ventricle. In contrast to Cx43, Cx40 it is not expressed within the muscular structures of the interventricular septum. However, Cx40 is expressed in the His-Bundle and the bundle branches. Cx43 is seen in both ventricles and the interventricular septum in early development and later on also in the atrial compartments. In the conduction system Cx43 is not represented (Delorme et al., 1997; Alcoléa et al., 1999). Cx45 is in early cardiogenesis the only connexin expressed in all cardiac compartments and its loss resulted in conduction block and cushion defects with lethal outcome at embryonic day 10 (Kumai et al., 2000; Nishii et al., 2001). During further morphogenesis it is progressively down-regulated and absent in the adult mouse ventricle. The expression pattern of connexins during development of the human heart is less well examined, but the connexin labeling pattern in human fetal heart resembles that of the mouse heart, with the exception that in the atria of human fetal hearts Cx43 is only expressed at very low levels whilst in the mouse heart it is abundant (Kaba et al., 2001; Coppen et al., 2003). Thus, although comprehensive analyses are still missing, at least to some extent connexin expression pattern in the mouse heart parallels that of human fetal hearts.

Furthermore, to stress the importance that not only the correct temporal expression of a connexin is essential for normal heart growth but also the level of connexin expression, studies in mice revealed that both homozygous Cx43 knock-out and Cx43 over-expression may result in outflow tract obstruction and conotruncal cardiac malformations (Reaume et al., 1995; Ewart et al., 1997). This, demonstrates the importance of an exact regulation of Cx43-expression for normal heart growth. What is most interesting is that in a mouse model a point mutation in the Cx43 gene led to a reduction in Cx43 expression, a reduction in gap junction coupling and a disruption of gap junction plaque assembly. These changes were accompanied by cardiac defects like patent foramen ovale, enhanced fibrosis, worsening of right and left ventricular function, and also conduction anomalies. Extracardial changes resembled the syndrome of oculodentodigital dysplasia (ODDD; Flenniken et al., 2005). This inborn syndrome is also seen in man and up to today over 60 mutations in the Cx43 gene have been described associated with this syndrome. Unlike the situation in mice, human cardiac malformations are not very common in ODDD. In man, only in few cases of ODDD conduction anomalies (atrioventricular block, right bundle branch block) and the appearance of VSDs and pulmonary stenosis have been described (Paznekas et al., 2003, 2009).

Moreover, a study with Cx40 knock-out mice demonstrated the importance of this connexin in generation of the mature apex to base activation of the heart (Sankova et al., 2012). Additionally, Kirchhoff et al. (2000) reported on various mice knock-out models with either Cx40 and/or Cx43 homozygous or heterozygous ablation with the occurrence of conduction anomalies and cardiac malformations (ASDs or VSDs, myocardial hypertrophy). In their study, the authors also found out that Cx43 haploinsufficiency even impaired the morphological phenotype of Cx40 deficiency. This was confirmed by another study of Simon et al. (2004) who detected malformed ventricles with an abnormal orientation in Cx40 and Cx43 double knock-out mice.

Conotruncal malformations, including TAC (birth prevalence 0.011%), double outlet right/left ventricle (birth prevalence 0.016%), Tetralogy of Fallot (TOF; birth prevalence 0.042%), and TGA (birth prevalence 0.032%; van der Bom et al., 2012), typically show malformations of the cardiac outflow tract. The critical time frame for the origination of conotruncal heart defects seems to be the fifth week of human embryonic development. At this specific moment in time the common outflow tract of the right and left heart, the Truncus arteriosus, is divided by a spiral shaped septum which ensures the correct association of the great arteries to the corresponding ventricles. Depending on the nature of malformation of the aorticopulmonary septum the afore mentioned cardiac defects may occur: a complete absence of the septum results in the development of TAC (common arterial trunk), lack of septal spiralization leads to TGA and anterior malalignment of the septum together with incomplete closure to double outlet right/left ventricle or TOF.

Hence, it is conceivable that the genesis of these cardiac defects might have the same etiopathologic origin and since the connexins play an important role in cardiac development it might be possible that certain alterations in temporal or spatial distribution of the main cardiac connexins may be at least co-responsible for conotruncal malformations. On the other side, it may also be imagined that conotruncal malformations themselves may secondarily lead to alterations in connexin expression and distribution.

Nearly 20 years ago, Britz-Cunningham et al. (1995) analyzed Cx43 mutations in patients with cardiac malformations and defects of laterality and they found missense mutations in parts of the Cx43 gene encoding for consensus phosphorylation sites within the Cx43 C-terminus. The authors could demonstrate that these mutations altered Cx43 phosphorylation by PKA or PKC, both protein kinases known to be important for Cx43–intercellular communication.

With these very interesting and new observations the authors started a world-wide discussion on whether or not Cx43 mutations are responsible or at least partly responsible for the development of cardiac malformations.

Therefore, the aim of this review is to discuss today’s knowledge of connexin alterations in human conotruncal heart defects and the possible impact of connexins on the development of the same.

## TRUNCUS ARTERIOSUS COMMUNIS

**FIGURE 2:**
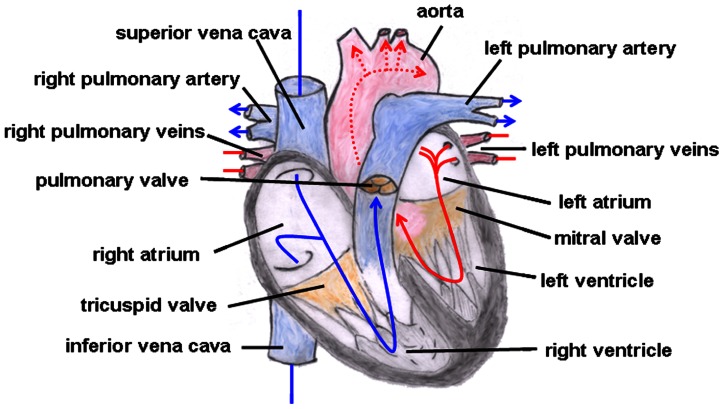
**Normal cardiac anatomy.** The blue arrows give the flow of oxygen-poor blood from both caval veins via the right atrium and the right ventricle to the left and right pulmonary artery. The red arrows give the flow of oxygen-rich blood from the pulmonary veins via the left atrium and the left ventricle to the aorta.

**FIGURE 3:**
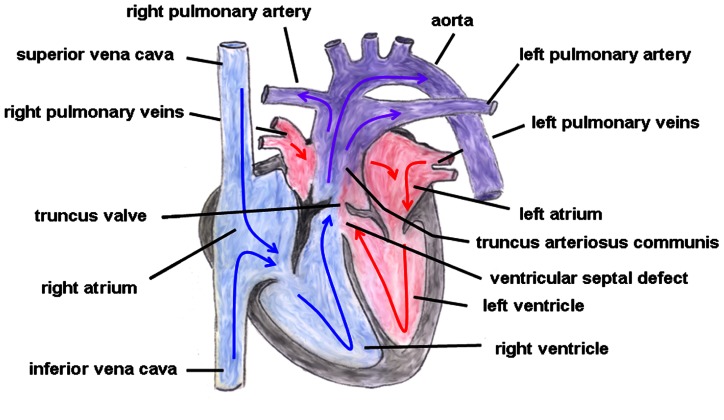
**Truncus arteriosus communis.** Both great arteries (i.e., pulmonary artery and aorta) emerge together from a solitary arterial trunk. The blue arrows give the flow of oxygen-poor blood from both caval veins via the right atrium and the right ventricle and via the truncus valve to the Truncus arteriosus communis. The red arrows give the flow of oxygen-rich blood from the pulmonary veins via the left atrium and the left ventricle to the Truncus arteriosus communis.

Truncus arteriosus communis can be classified into three different types depending on the outflow of the pulmonary artery: type 1 (rare): pulmonary trunk originates from the lateral wall of the TAC and branches into one left and one right pulmonary artery; type 2 (common type): two pulmonary arteries with proximate origins separately branch off from the posterolateral aspect the common arterial trunk; type 3 (rare): both pulmonary arteries originate separately from the left and right lateral side of the common trunk. The symptoms of this heart defect include right heart hypertrophy, pulmonary overflow with consecutive pulmonary hypertension, Eisenmenger’s syndrome, and heart failure (**Figure 2** gives the normal cardiac anatomy, in **Figure 3**, TAC type 3 is depicted).

The clinical picture of TAC is often associated with the DiGeorge syndrome (2q11 microdeletion, conotruncal heart malformations, thymic and parathyroid hypoplasia, cleft palate, and facial dysmorphism). This inborn malady is evoked by disturbances in the migration of the cardiac neural crest cells. Kirby et al. (1983) have demonstrated in their seminal study done in chick embryos that ablation of a certain portion of the neural crest cells resulted in conotruncal heart defects including common arterial trunk. More recently, Huang et al. (1998a, b) could show in mice embryos that alterations in Cx43 gene dosage also resulted in conotruncal heart defects with pathologic morphology of the right ventricle, thinning of the myocardium and narrowing of the right ventricular outflow tract (RVOT). Moreover, they could demonstrate that gap junctional communication was increased in neural crest cells of Cx43 overexpressing mice and decreased in those of Cx43 knock-out mice. This phenomenon was accompanied by corresponding migration changes in the cardiac neural crest cells and by a perturbation of cardiomyocyte proliferation. Thus, the authors concluded that intercellular communication in cardiac neural crest cells might be important for the myocardialization of the ventricular outflow tract, i.e., the regular shaping of the outflow septum. The impact of Cx43 on the development of the aorticopulmonary septum was underlined by a study of Waller et al. (2000) who also found that gain or loss of Cx43 function results in outflow tract anomalies and preferentially in persistent arterial trunk. In addition, induction of mutagenesis with *N*-ethyl-*N*-nitrosourea in mice revealed that in the Cx43 gene a G to A substitution which generated a premature stop codon was sufficient to cause conotruncal malformation and coronary aneurysms (Yu et al., 2004). In addition to this result, Yu et al. (2004) also reported on a point mutation within another protein, the semaphorin, which resulted in common arterial trunk and interrupted aortic arch. Since the semaphorin family is supposed to give environmental cues for the migration of neural crest cells (Brown et al., 2001) it seems clear that perturbation of neural crest cell migration might hinder the correct formation of the arterial trunk. This viewpoint is also supported by the excellent review of Hutson and Kirby (2003), who pointed out that aortic arch formation and outflow tract septation deeply depend on a correct deployment of cardiac neural crest cells. However, Cx43 is obviously not the only responsible factor for cardiac neural crest migration: there are several other protein families including growth factors [i.e., fibroblast growth factor, bone morphogenetic protein (BMP) or Wnt signaling pathway] as well as transcription factors (i.e., AP2, Sox9, FoxD) or adhesion molecules like N-cadherin being responsible for cardiac neural crest development. These factors and pathways are outlined in the comprehensive review of Kirby and Hutson (2010). Therefore, it can be concluded from the aforesaid that disturbances in Cx43-expression are very important factors in the pathogenesis of TAC but not the sole ones.

There are no experimental studies existing on the potential influence of Cx40 or Cx45 on the development of persistent arterial trunk.

## TRANSPOSITION OF THE GREAT ARTERIES

**FIGURE 4:**
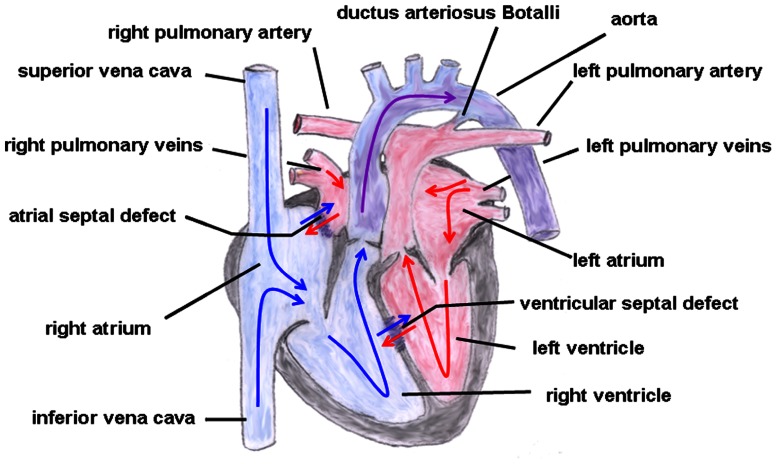
**Transposition of the great arteries.** The ventriculoarterial discordance with the pulmonary artery connected to the left and the aorta connected to the right ventricle is shown. Also depicted is an atrial and ventricular septal defect and a patent ductus arteriosus Botalli. The blue arrows give the flow of oxygen-poor blood from both caval veins via the right atrium and the right ventricle to the aorta. The red arrows give the flow of oxygen-rich blood from the pulmonary veins via the left atrium and the left ventricle to the pulmonary artery.

The hallmark of this cardiac malformation is the ventriculoarterial discordance in such a way that the aorta arises from the morphological right ventricle and the pulmonary artery from the morphological left ventricle with both main vessels remaining parallel to each other (i.e., they do not cross over, which would be the normal condition; Martins and Castela, 2008; **Figure 4**). Moreover, atypical origins of the coronary arteries and VSDs or ASDs are not uncommon. Depending on whether the aorta is located on the right or left side of the pulmonary artery this cardiac malformation is sub-classified into d(exter)- or l(aevus)-transposition, respectively. Additionally, a distinction can be made between complete transpositions, i.e., concordant atrioventricular and discordant ventriculoarterial connections (the atria are connected to their corresponding ventricles but the great arteries emerge from the “wrong” ventricle) and congenitally corrected transpositions with atrioventricular and ventriculoarterial discordance [the right atrium (influx of venous blood) is connected to the left ventricle from which the pulmonary artery originates and the left atrium (influx of arterial blood) is coupled to the right ventricle from which the aorta emerges]. In the first condition, both circuits (systemic and pulmonary circulation) run in parallel resulting in a severe hypoxemic status in which the survival depends on the adequate mixing between the two circulations be it via a VSD or ASD or a patent ductus arteriosus Botalli, whereas in the second condition (systemic and pulmonary circulation run in series) patients could remain asymptomatic over a longer period of time.

The exact etiology of this cardiac defect still remains undetected. However, some risk factors like maternal diabetes mellitus (Lisowski et al., 2010), herbicides (Loffredo et al., 2001), maternal use of antiepileptic medication (Thomas et al., 2008) have been identified as well as some cases associated with the DiGeorge syndrome (Laitenberger et al., 2008). Although neural crest cell migration plays a significant role in the pathogenesis of conotruncal heart defects, surprisingly, TGA could not be detected experimentally after neural crest ablation (Kirby, 2002). Interestingly enough, Costell et al. (2002) presented a mouse model of perlecan (heparan sulfate proteoglycan 2) null mutation exhibiting the cardiac phenotype of complete TGA, and the authors concluded from their study that perlecan has a role in the organization and differentiation of the outflow tract mesenchyme. Additionally, the highly mutagenic agent *N*-ethyl-*N*-nitrosourea also had the ability to induce TGA in mice, but more commonly TAC and outflow tract anomalies together with coronary aneurysms were seen (Yu et al., 2004). Whether this Cx43 point mutation described in the study of Yu et al. (2004), which was found together with outflow tract anomalies, might also account for the heart defect “transposition of the great arteries” still remains to be elucidated. However, it was shown in a very recent study on human myocardial anomalies including TGA that N-cadherin and Cx43 expression and distribution was not altered in both left and right ventricle as compared to normal hearts (Mahtab et al., 2012). Although in this study the number of cases is considerably small (only three hearts were examined) the authors could clearly show that Cx43 together with N-cadherin accrued at the intercalated disks with a normal pattern. To date, there are no studies available describing pathological alterations of Cx40, Cx43, or Cx45 having a causal relationship in the development of TGA. However, in a study of Haefliger et al. (2000) done in mice the influence of another connexin, the Cx37, on this cardiac defect was examined. This connexin isoform, expressed in larger quantities in endothelial cells in a number of vessels and only very sparsely in cardiomyocytes, seems to play a not unimportant role in conotruncal development. Mice treated with all-trans retinoic acid during early embryonic life exhibited complete transposition of aorta and pulmonary arteries. Ventricles of these mice showed an abnormal high expression of Cx37, but whether the expression level of Cx37 is really a co-factor for the heart defect TGA is not finally clarified, as it seems so that the Cx37 over-expression was not directly linked to that cardiac malformation (Haefliger et al., 2000). Nevertheless, it should be taken into account that Cx37 typically is expressed in vascular endothelium and thus the described malformations may also be influenced by changes in angiogenesis or vascular function. However, this is unclear at present.

## DOUBLE OUTLET RIGHT VENTRICLE

**FIGURE 5:**
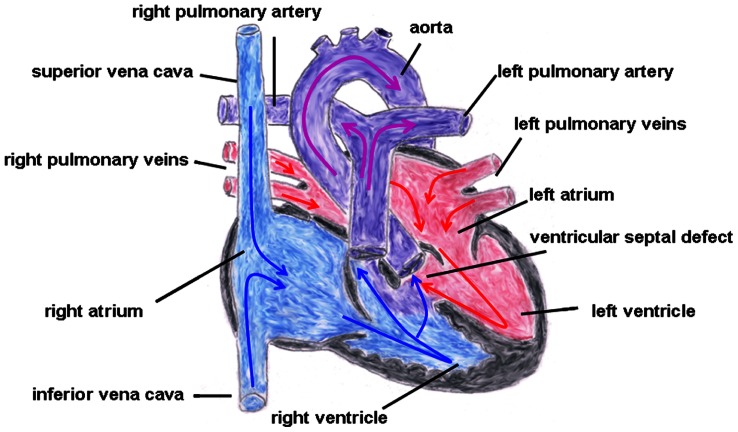
**Double outlet right ventricle (VSD-type).** Origination of both great arteries (aorta and pulmonary artery) from the right ventricle with a concomitant ventricular septal defect. The blue arrows give the flow of oxygen-poor blood from both caval veins via the right atrium and the right ventricle to the pulmonary artery and aorta. The red arrows give the flow of oxygen-rich blood from the pulmonary veins via the left atrium, the left ventricle to the aorta.

The characteristic of this heart defect is the origination of both great arteries (aorta and pulmonary artery) from the right ventricle (shown in **Figure 5**). This malformation occurs in variable forms depending on the degree of malposition of the great arteries, the location of the concomitant VSD and the occurrence of RVOT obstruction. Thus, a distinction can be made between a VSD-type double outlet right ventricle (DORV; VSD with sub-aortic location), Fallot-type DORV (VSD with sub-aortic location and pulmonary stenosis) and TGA-type DORV (VSD with sub-pulmonary location, Taussig–Bing syndrome; Artrip et al., 2006). Therefore, depending on the exact morphology of the DORV, the clinical manifestation is different: DORV physiology might resemble a large unrestrictive VSD, a TOF or a TGA. This heterogeneity also implies different operative approaches and the timing of surgical repair. Associated cardiac malformations include a juxtaposition of atrial appendages, obstruction of the aortic arch, mitral atresia, or atrioventricular septal defects. Moreover, extracardiac manifestations can be found, such as heterotaxia, esophageal atresia, exomphalos, and chromosomal anomalies like the trisomy 18 (Tennstedt et al., 1999). An involvement of the neural crest zone in the development of DORV has been described, but as outlined in the comprehensive review of Obler et al. (2008) disturbances in neural crest cell migration might not be the unique reason for the formation of a DORV.

About 10 years ago, Gu et al. (2003) published a study about Cx40 homozygous null mice, showing that if Cx40 is completely knocked-out cardiac malformations like DORV or TOF occurred. However, given the fact that only 30% of the Cx40^–^^/^^–^ mice exhibited cardiac malformations the authors suggested that down-regulation of Cx40 in the neural crest zone might play an indirect role in the development of the described heart defects. Moreover, a very recent study in mice revealed that if Hand2 (a transcription factor involved in the morphogenesis of limbs), which is expressed in cardiac neural crest cells and also in the right ventricle and outflow tract, is deleted outflow tract anomalies, i.e., DORV and VSD occurred (Holler et al., 2010). The authors also found that Cx40, which is expressed in neural crest cells of wild-type mice, was significantly reduced in the Hand2 knock-out mice and they concluded from their experiments that Hand2 directly binds to the Cx40 promoter, thereby enhancing Cx40 expression, and that intercellular communication is a critical part for a proper outflow tract development.

A human study focusing on possible mutations of Cx43 in the development of cardiac defects was done on cardiac material of aborted fetuses, and the authors reported on eight Cx43 mutations which they found in a heart with DORV (Chen et al., 2005). These mutations were in the C-terminus near the transmembrane region and one mutation (the Pro283Leu missense mutation) was thought to limit Cx43 degradation. As the amount of Cx43 is important for the development of the conotruncus the authors concluded that this point mutation might be involved in the occurrence of DORV.

## TETRALOGY OF FALLOT

**FIGURE 6:**
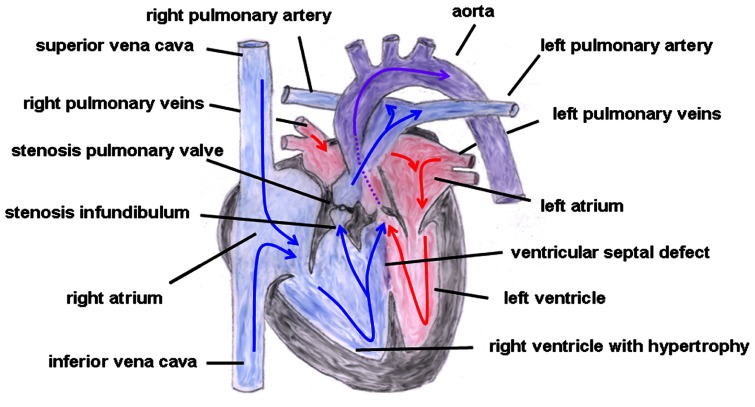
**Tetralogy of Fallot.** Depicted are the four cardiac malformations described by Fallot: (1) right ventricular hypertrophy, (2) pulmonary stenosis (valvular and infundibular), (3) ventricular septal defect with (4) overriding of the aortic root. The blue arrows give the flow of oxygen-poor blood from both caval veins via the right atrium and the right ventricle to the pulmonary artery and via the ventricular septal defect to the aorta (right–left shunt). The red arrows give the flow of oxygen-rich blood from the pulmonary veins via the left atrium and the left ventricle to the aorta.

In the year 1888, the French physician Étienne-Louis Arthur Fallot described a congenital heart defect with four cardiac malformations: (1) right ventricular hypertrophy, (2) pulmonary stenosis (valvular or infundibular), (3) VSD with (4) overriding of the aortic root (**Figure 6**). This congenital heart anomaly is the most frequent inborn cyanotic heart disease. Nowadays, in infants with Morbus Fallot primary repair is done in early childhood, mostly below the age of 1 year, and the prognosis of these patients is excellent with most of them reaching adulthood (Shinebourne et al., 2006; Christian et al., 2009). Associated with Morbus Fallot anomalies of the coronary arteries, ASDs, a right aortic arch and also extracardial anomalies like gastrointestinal or facial anomalies might exist (Piran et al., 2011). TOF typically occurs sporadically, but also familiar recurrences have been described. The etiology is multifactorial, but maternal diabetes mellitus, maternal intake of retinoic acid, and phenylketonuria have been reported as risk factors (Bailliard and Anderson, 2009). Moreover, an association with chromosomal anomalies which include trisomy 13, 18, and 21 exist. Especially the coincidence of Morbus Fallot together with 22q11.2 microdeletion is not infrequent. Maeda et al. (2000) reported in a study that of 212 Japanese patients 28 patients (13%) had 22q11.2 microdeletion and that all of these 28 patients had at least one extracardiac abnormality. Thus, the authors concluded that, at least in syndromic Morbus Fallot, 22q11.2 microdeletion is quite common (Maeda et al., 2000), although its pathogenetic role is still unclear. Moreover, some studies involving non-syndromic TOF described dominant mutations in genes encoding for various transcription factors, like GATA4, Nkx2.5, or ZFMP2/FOG2 (Goldmuntz et al., 2001; Nemer et al., 2006; De Luca et al., 2011).

Given the accepted fact that regular Cx43 expression is important for neural crest development (Waldo et al., 1999) the question arises if mutations in the Cx43 gene might also be responsible for the occurrence of Morbus Fallot. A very early publication on this subject was done by Reaume et al. (1995), who created a Cx43 knock-out mouse and could demonstrate that these mice had outflow tract anomalies and died soon after birth. However, the full picture of Morbus Fallot was not seen, suggesting that additional factors might be required. Few years later Sullivan et al. (1998) presented a study in which they used a dominant negative strategy to reduce gap junctional coupling within the cardiac neural crest cells. They could also demonstrate that outflow tract obstruction, right ventricular hypertrophy, and abnormal bulging of the right ventricle occurred, but the full syndrome of Morbus Fallot was not observed.

One recent study seemed to confirm the findings of Britz-Cunningham et al. (1995) on mutations in the C-terminus tail of Cx43 in patients with inborn cardiac defects (Wang et al., 2010). In the analysis of Wang et al. (2010) over 400 Chinese children were included. Most of these children had VSDs or ASDs or Morbus Fallot, and three heterozygous missense mutations in the C-terminus were found in all these patients with congenital heart defects, whereas no mutations were detectable in the normal control subjects. As the C-terminus includes important phosphorylation sites (**Figure 1**) the authors concluded that the mutations could have an impact on the structure of Cx43 and on normal channel function. In contrast, 1 year later another very interesting study came to a radically opposed conclusion: in this study, 300 patients with conotruncal heart defects including Fallot’s Tetralogy were investigated and the authors discovered two silent mutations in the Cx43 gene in eight patients, but no mutations were found which would alter Cx43 amino acid sequence (Huang et al., 2011). Moreover, this working group constructed a mouse model with homozygous or heterozygous mutations of serine residues known to be targeted by PKC or CK1 (casein kinase 1), both enzymes being important for channel conductance and the assembly of Cx43 gap junction channels. Surprisingly, in the hearts of these mice, which were viable with a normal life-span, no changes in Cx43 distribution or expression, and no congenital heart defects, were detected. The authors concluded that Cx43 does not contribute to a large extent to the development of heart malformations. Thus, the question of whether or not mutation in Cx43 gene is involved in the origination of Morbus Fallot remains unsolved.

Another study group analyzed Cx43 expression and distribution in patients with Morbus Fallot and they found that in these patients Cx43, which is normally confined to the poles of the cardiomyocytes, was distributed around the entire circumference of the cells. Moreover, total Cx43 staining, analyzed by a fluorescence activated cell sorting (FACS)-approach, was significantly lower in the patient group having Morbus Fallot compared to the control group (patients with VSDs; Kołcz, 2002, 2005). The authors concluded from their studies that these connexin alterations might be responsible for cardiac arrhythmias, seen frequently in patients with Morbus Fallot and that changes in Cx43 localization might contribute to a dysfunction of the right ventricle. However, it should be noted that histological analyses were not carried out on the intact heart tissue but on single cardiomyocytes cultured in Petri dishes. Since Cx43 has a very short half-time it is not unlikely that the described Cx43 re-distribution is more due to the culture conditions than to the heart defect or a more complex response of somehow altered cardiomyocytes to the culture conditions. In the end, the precise role of Cx43 in the development of Morbus Fallot cannot be assessed.

Given the information that the other important connexin, Cx40, is also involved in cardiac morphogenesis, several working groups have analyzed this connexin in relation to the occurrence of TOF. These studies done in man revealed that Morbus Fallot was associated with above-average frequency with copy number variants in the Cx40 gene (Greenway et al., 2009; Soemedi et al., 2012). Moreover, it was shown by Greenway et al. (2009) that in TOF patients Cx40 expression in RVOT, which is malformed in Fallot’s Tetralogy, was enhanced. This finding could indicate that Cx40 might be among the disease genes involved in the occurrence of Morbus Fallot.

Since mutations in the C-terminus of Cx43 have been found in TOF patients, another interesting aspect to consider would be if the C-terminal end of Cx40 might also be mutated in patients with Morbus Fallot. A very interesting study published this year (Guida et al., 2013) characterized over 150 patients with non-syndromic Fallot and found in 1% of their patients a heterozygous nucleotide change in the Cx40 gene leading to altered amino acid sequence. This Pro265Ser variant was not seen in healthy volunteers [amino acid 265 is the binding region for src (sarcoma Rous kinase)]. Further experiments of this working group on the cellular level revealed that this mutant Cx40 led to a reduced gap junctional coupling. In addition, introducing the Pro265Ser mutant of Cx40 into zebrafishes also was associated with malformations of the heart tube.

However, a definite clarification whether connexins are causally involved in the development of Morbus Fallot or whether their change in expression is an epiphenomenon is still pending.

## POSSIBLE MOLECULAR MECHANISMS

The role of connexins in the development of cardiac malformation has emerged over the past decade by introducing the knock-in and knock-out mice and a lot of research has been done in this field. However, still only little is known about the signaling pathways in these mice to unravel how connexin alterations might be linked to the origination of conotruncal heart defects. By contrast, some studies have been published which used an approach from a different perspective. Dupays et al. (2005) described in their study results on Nkx2.5-deficient mice. They found out that in homozygous Nkx2.5 null mice embryos Cx40, Cx43, and Cx45 could not be detected within the myocardium, whereas these connexins were still detectable in other organs. Moreover, although 75% of these embryos had a normal sequence of cardiac activation and conduction along the conduction system, heart rate was very slow. In the other remaining 25% of mutant mice the ventricle was the first chamber being activated thus showing a reverse action of cardiac excitation. The Nkx2.5 null mice embryos also exhibited an insufficient development of the ventricular trabeculae. Besides, heart malformations like ASDs and VSDs have been seen although complex cardiac malformations could not be detected maybe because of premature death occurring in theses mice (Terada et al., 2011). However, in man Nkx2.5 mutations are also associated with cardiac malformations like ASDs and VSDs and additionally with other more complex cardiac malformations (Morbus Fallot, pulmonary atresia; Schott et al., 1998; Wang et al., 2011). However, for obvious reasons analysis of connexin expression could not be done in the patients hearts.

Another transcription factor which has been described in the context of the Holt–Oram syndrome is the T-box transcription factor Tbx5 (Basson et al., 1997). Patients suffering from this inborn syndrome have malformed limbs and cardiac defects like ASD, VSD, and also more severe cardiac malformations. In a mouse model of heterozygous Tbx5 mutants Bruneau et al. (2001) could demonstrate that a phenotype similar to the Holt–Oram syndrome occurred, and that interestingly Cx40 gene expression was down-regulated in the atria and ventricles of these mice.

GATA4 is another transcription factor known to be associated with cardiac development. Recently, a point mutation of GATA4 was detected within a large cohort of kindred persons, who exhibited ASDs and in some cases also VSDs and abnormalities of the pulmonary valve (Garg et al., 2003). All these family members carried a glycine to serine substitution at position 296 of GATA4, which was accompanied by a diminished binding ability to the DNA consensus sequence and in addition by reduced interaction with Tbx5.

Consistent with all these findings Linhares et al. (2004) showed that in the Cx40 promoter binding sites exist for Tbx5, Nkx2.5, and GATA4. Both nuclear factors Nkx2.5 and GATA4 transactivate the Cx40 promoter thereby regulating Cx40 expression. This was compatible with the finding that in Nkx2-5 knock-out mice Cx40 expression was markedly down-regulated. On the other hand, the nuclear factor Tbx5 seemed to have a more repressive role on the Cx40 promoter, which was in contrast to the finding of Bruneau et al. (2001), who demonstrated the opposite. A possible explanation discussed by Linhares et al. (2004) would be that the promoter sequence used in their study was considerably shorter than that of Bruneau et al. (2001) therefore lacking important necessary elements.

Thus, besides connexin mutations seen in human cardiac malformations also mutations in transcription factors accompanied by altered connexin expression might have an impact on inborn heart defects.

However, a detailed description of how signaling pathways may be linked to cardiac malformations awaits further studies.

Nevertheless, a problem with these mice knock-out studies is that the finally resulting cardiac phenotype is not investigated with regard to the molecular mechanisms linking the knock-out-target to the cardiac development. Moreover, from a cardiological point of view it is remarkable that mutations or knock-out in very different genes very often result in ASDs or VSDs. This might indicate that between connexins, transcription factors like Nkx2.5, Tbx5, GATA4, etc. and cardiac malformations there might exist one (or more) missing links which still need to be elucidated.

## CONCLUSION

Taken together, until now there is no conclusive evidence that human inborn conotruncal heart defects are caused by mutations or changes in connexins. From the view point of cell biology it is tempting to speculate that a protein so deeply involved in intercellular communication, regulation of growth and differentiation as well as of cell cycle could be involved in the pathogenesis of malformation. However, although Cx43 knock-out mice can present malformation of the RVOT with some similarity to Morbus Fallot, this does not warrant the conclusion that Morbus Fallot results from Cx43 changes. There probably are many regulatory steps and proteins involved in cardiac organogenesis, so that the malfunction of each and any may cause a common phenotype. This leads to our present view that the pathogenesis of conotruncal human inborn heart defects seems multifactorial and that there is at present for the majority of inborn cardiac malformations no evidence for a direct or causal role of connexins. Although, studies in mice revealed that at least in some cases of cardiac defects a pathogenetic involvement of connexins might be evident, this does not exclude that other proteins with an impact on protein trafficking or membrane anchoring may be involved and may lead to secondary changes in connexins either as an epiphenomenon or playing an aggravating role. In addition, the cardiac defect could cause hemodynamic changes which would lead to altered local stretch. Since stretch has an effect on cytoskeleton and on the subcellular distribution of Cx43 (Salameh et al., 2010), this also may lead to secondary changes in connexin expression and localization. Whether such changes have a feedback effect on cellular growth and differentiation is unclear. In summary, a direct unifactorial role of connexins in human inborn conotruncal heart defect seems improbable, but a more complex role or a bystander effect is reasonable.

Not chicken, not egg, but complex bidirectional interactions between connexins and tissue developments and vice-versa.

## Conflict of Interest Statement

The authors declare that the research was conducted in the absence of any commercial or financial relationships that could be construed as a potential conflict of interest.
